# Characteristics and outcome of patients with newly diagnosed advanced or metastatic lung cancer admitted to intensive care units (ICUs)

**DOI:** 10.1186/s13613-018-0426-2

**Published:** 2018-08-04

**Authors:** C. Barth, M. Soares, A. C. Toffart, J. F. Timsit, G. Burghi, C. Irrazabal, N. Pattison, E. Tobar, B. F. Almeida, U. V. Silva, L. C. Azevedo, A. Rabbat, C. Lamer, A. Parrot, V. C. Souza-Dantas, F. Wallet, F. Blot, G. Bourdin, C. Piras, J. Delemazure, M. Durand, J. Salluh, E. Azoulay, Virginie Lemiale, Lung Cancer in Critical Care (LUCCA) Study Investigators, Célica Irrazábal, Pierina Bachetti, Vicente C. Souza-Dantas, Mauro M. Zamboni, Aureliano Sousa, Bruno F. C. Almeida, Lúcio S. Santos, Pedro Caruso, Ulysses V. A. Silva, Luciano C. P. Azevedo, Guilherme P. P. Schettino, Cláudio Piras, Stéphanie B. Piras, Albano S. M. T. Silva, Eduado Tobar, Nivia Estuardo, François Blot, Bruno Raynard, Antoine Parrot, Florent Wallet, Christian Lamer, Alexandre Duguet, Alexandre Demoule, Julie Delemazure, Julien Mayaux, Thomas Similowski, Michel Durand, Geraldine Dessertaine, Pr Jean François Payen, Anne-Claire Toffart, Jean-François Timsit, Gael Bourdin, Claude Guerin, Antoine Rabbat, Aurélie Lefebvre, Élie Azoulay, Natalie Pattison, Natalie Pattison, Gastón Burghi, Gastón Burghi, Gastón Burghi

**Affiliations:** 1 Medical ICU, AP-HP, Hôpital Saint-Louis, 1 Avenue Claude Vellefaux, 75010 Paris, France; 2 Post-Graduation Program, Instituto Nacional de Câncer, Rio de Janeiro Department of Clinical Research, D’Or Institute for Research and Education, Rio de Janeiro, Brazil; 3 Inserm, u 823, Institut A Bonniot, Grenoble, France; 4 Medical ICU, Hôpital Bichat-Claude Bernard, Paris, France; 5 ICU, Hospital Maciel, Montevideo, Uruguay; 6 ICU, Instituto Medico Especializado Alexander Fleming, Buenos Aires, Argentina; 7 ICU, Royal Brompton NHS Foundation Trust, London ICU, Royal Marsden Hospital, London, UK; 8 ICU, Hospital Clinico Universidad de Chile, Santiago, Chile; 9 ICU, Hospital A. C. Camargo, São Paulo, Brazil; 10 ICU, Fundação Pio XII-Hospital do Câncer de Barretos, Barretos, Brazil; 11 ICU, Hospital Sírio Libanês, São Paulo, Brazil; 12 Thoracic ICU, Hôpital Cochin, Paris, France; 13 ICU, Institut Mutualiste Montsouris, Paris, France; 14 Medical ICU, Hôpital Tenon, Paris, France; 15 ICU, Instituto Nacional de Câncer-Hospital do Câncer I, Rio de Janeiro, Brazil; 16 Medical-Surgical ICU, Hospices Civils de Lyon Centre Hospitalier Lyon Sud, Lyon, France; 17 ICU, Institut Gustave Roussy, Villejuif, France; 18 Medical ICU, Hôpital de la Croix-Rousse, Lyon, France; 19 ICU, Vitória Apart Hospital, Vitória, Brazil; 20 Medical ICU, Groupe Hospitalier Pitié Salpêtrière, Paris, France; 21 Surgical ICU, Hôpital A. Michallon Chu de Grenoble, Grenoble, France

**Keywords:** Lung cancer, Metastatic, Outcome, Intensive care, Chemotherapy

## Abstract

**Background:**

Although patients with advanced or metastatic lung cancer have poor prognosis, admission to the ICU for management of life-threatening complications has increased over the years. Patients with newly diagnosed lung cancer appear as good candidates for ICU admission, but more robust information to assist decisions is lacking. The aim of our study was to evaluate the prognosis of newly diagnosed unresectable lung cancer patients.

**Methods:**

A retrospective multicentric study analyzed the outcome of patients admitted to the ICU with a newly diagnosed lung cancer (diagnosis within the month) between 2010 and 2013.

**Results:**

Out of the 100 patients, 30 had small cell lung cancer (SCLC) and 70 had non-small cell lung cancer. (Thirty patients had already been treated with oncologic treatments.) Mechanical ventilation (MV) was performed for 81 patients. Seventeen patients received emergency chemotherapy during their ICU stay. ICU, hospital, 3- and 6-month mortality were, respectively, 47, 60, 67 and 71%. Hospital mortality was 60% when invasive MV was used alone, 71% when MV and vasopressors were needed and 83% when MV, vasopressors and hemodialysis were required. In multivariate analysis, hospital mortality was associated with metastatic disease (OR 4.22 [1.4–12.4]; *p* = 0.008), need for invasive MV (OR 4.20 [1.11–16.2]; *p* = 0.030), while chemotherapy in ICU was associated with survival (OR 0.23, [0.07–0.81]; *p* = 0.020).

**Conclusion:**

This study shows that ICU management can be appropriate for selected newly diagnosed patients with advanced lung cancer, and chemotherapy might improve outcome for patients with SCLC admitted for cancer-related complications. Nevertheless, tumors’ characteristics, numbers and types of organ dysfunction should be taken into account in the decisional process before admitting these patients in ICU.

## Background

Lung cancer is the most frequent malignancy worldwide with an incidence of 1.8 million new cases a year in 2012 and the most common cause of death from cancer [1]. The development of targeted therapies and the emergence of immunotherapy [2, 3] recently improved outcome for patients with advanced and metastatic non-small cell lung cancer. However, those patients remain exposed to numerous complications related to cancer itself, to treatments and to other comorbidities, and in many cases, require admission to intensive care units (ICUs) for their management.

ICU admission for cancer patients has been considered futile for a long time due to high mortality rates [4]. Lung cancer patients were particularly judged as poor candidates for ICU admission because their prognosis was thought to be even worse than other cancer patients [5]. However, improvement of the prognosis in ICU has been reported for patients with solid tumors over the last decades [6]. Taccone et al. [7] found that the mortality rate of cancer patients was similar as the general population. Other studies showed that cancer patients had mortality rates equivalent to patients with severe comorbidities like cardiac failure or cirrhosis [8, 9].

The main factors associated with mortality, such as acute respiratory failure [10–13], sepsis [10, 14, 15], more than two organ dysfunctions [12, 14, 15], the need for mechanical ventilation (MV) [10, 14–17], the need for vasopressors [15, 17, 18], a performance status ≥ 2 [11, 13, 18] and metastatic [17] or progressive disease [12] have been assessed for all lung cancer patients. However, for patients with newly diagnosed lung cancer, factors associated with outcome have not yet been described.

The aim of our study was to evaluate the prognosis in ICU, at hospital, at 3 and 6 months of newly diagnosed unresectable lung cancer patients.

## Patients and methods

### Design of the study

This retrospective observational cohort study analyzed the medical records of lung cancer patients who were admitted to the ICU between January 2010 and December 2013, using two databases from twenty-one European and South American ICU. All centers are listed in “Appendix.” These two databases were the Lung Cancer in Critical Care (LUCCA) [19] database and the Saint-Louis Hospital’s database for patients admitted to ICU. For patients from LUCCA database, the study was initially approved by the Brazilian National Ethics Committee (approval number CONEP 15.790) and subsequently by local and national ethics committees in the participating centers and countries. For patients from Saint-Louis Hospital, the ICU database was approved by the institutional review board (CECIC Clermont-Ferrand-IRB n5891; Ref: 2007-16), which waived the need for signed informed consent of the participants, in accordance with French legislation on noninterventional studies.

### Inclusion criteria, data collection

All patients aged over 18 years with a diagnosis of lung cancer admitted during the first month of diagnosis to the participating center’s ICUs could be included. Inclusion criteria included a histologically proven lung cancer staged as locally advanced or metastatic. Patients admitted for postoperative care were excluded from the analysis.

The following variables were collected at admission: age, gender, medical background, time since diagnosis, main admission reason. Oncologic characterization was also collected and included the histological type, the extension of the disease (metastatic versus non-metastatic), the potential preview anticancer treatments (chemotherapy, radiotherapy) and the pre-ICU (within the weeks before hospital admission) Eastern Cooperative Oncology Group performance status (ECOG-PS) [20].

The severity of the illness was evaluated using the Sequential Organ Failure Assessment (SOFA) score [21] and the Simplified Acute Physiology Score II (SAPS II) [22] at admission. Comorbidities were determined with the Charlson Comorbidity Index (CCI) [23].

ICU’s interventions were defined by the use of MV, including noninvasive ventilation (NIV) and invasive mechanical ventilation (iMV), the use of vasopressors, hemodialysis and oncologic treatments. The decisions to withdrawal/withhold life-sustaining therapies (WLTs) were also collected.

The primary outcome was hospital mortality. Secondary outcomes were ICU mortality, 3- and 6-month mortality. Also, patients who received chemotherapy during ICU stay were described.

### Statistical analysis

All data are presented as frequencies (percentage) for qualitative variables and medians (25th–75th percentiles) for quantitative variables. The variable of interest for outcome was hospital mortality. First, a univariate analysis was performed to compare patients who survived and patients who died during hospital stay, using nonparametric Wilcoxon test or Chi-square test, as appropriate. A logistic regression analysis was performed to identify independent prognostic variables among six characteristics of patients during ICU stay and ICU interventions (metastatic disease, chemotherapy during ICU, need and mode of mechanical ventilation, need of vasopressor). Two-sided *p* values < 0.050 were considered significant. The subgroup of patients who received chemotherapy during ICU stay was described. No comparisons were made in this subgroup of interest. Survival curves at 6 months were plotted using the Kaplan–Meier method. All statistical analyses were performed with Statview (SAS Institute Inc, USA).

## Results

### Patients’ characteristics

**Fig. 1:**
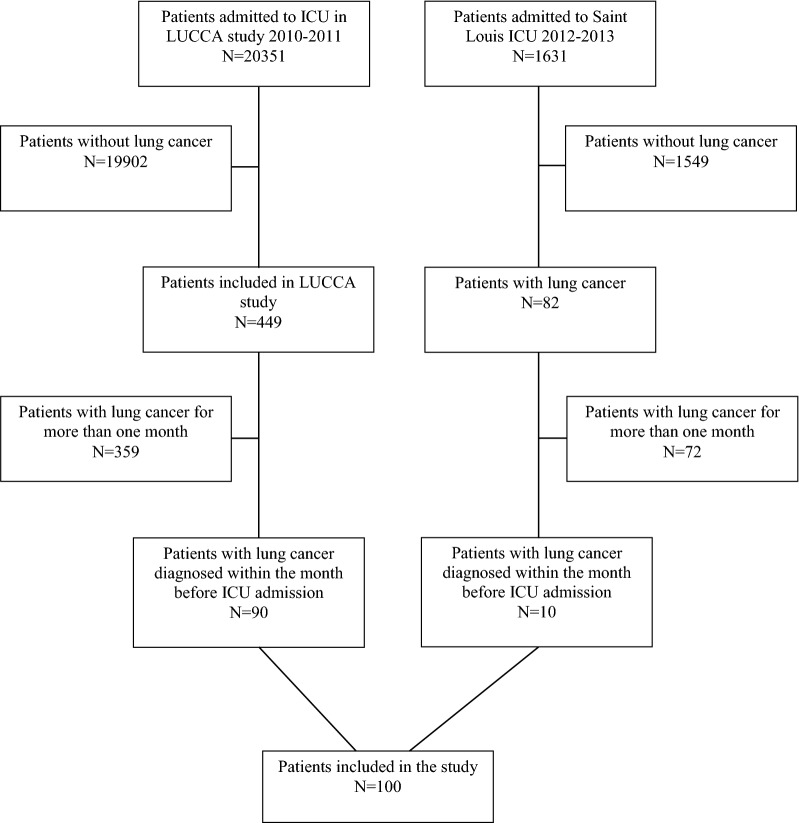
Flowchart

From January 2010 through December 2013, 100 patients admitted in ICU met the inclusion criteria (Fig. 1). Patients’ characteristics are summarized in Table 1.

**Table 1:** Patients’ characteristics at ICU admission

Characteristics	*n* (total = 100)
Age, in years median [IQR]	64 [56.0–72.0]
Male gender	68
Comorbidities	
COPD	13
Hypertension	43
Diabetes	18
Cirrhosis	2
Angina	5
Arrhythmia	13
DVT	5
Stroke	5
Malnourishment	11
Days since diagnosis, median [IQR]	7 [0–20.0]
Histological type	
SCLC	30
Adenocarcinoma	28
Squamous cell carcinoma	25
Large cell carcinoma	5
Other	12
Metastatic disease	70
ECOG-PS	
Low (0–1)	75
High (≥ 2)	24
Unknown	1
SOFA score, points, median [IQR]	8 [4.0–12.0]
SAPS II, points, median [IQR]	52 [41.0–64.0]
CCI, points, median [IQR]	0.5 [0–3.0]

*IQR* interquartile range, *COPD* chronic obstructive pulmonary disease, *DVT* deep vein thrombosis, *SCLC* small cell lung cancer, *ECOG-PS* Eastern Cooperative Oncology Group performance status, *SOFA* Sequential Organ Failure Assessment, *SAPS II* Simplified Acute Physiology Score, version II, *CCI* Charlson Comorbidity Index

The median time between cancer diagnosis and ICU admission was 7 days [0–20.0 days]; 31 patients had their diagnosis confirmed during their ICU stay.

Seventy-five percent of the patients had a good performance status (ECOG-PS = 0–1) before ICU admission. At admission, medians of SOFA, SAPS II and CCI scores were, respectively, of 8 [4.0–12.0], 52 [41.0–64.0] and 3 [3–6].

The main reasons for admission in ICU were acute respiratory failure (except from septic cause) (*n* = 46), septic shock (*n* = 40), cardiogenic shock (*n* = 4), coma (*n* = 4), cardiac arrest (*n* = 2) and miscellaneous reasons (*n* = 4). Among these admission reasons, 74% of patients presented with one or more lung cancer-related complication (37 with airway obstruction, 27 with pleural infusion, 13 with superior vena cava syndrome, 8 with pericardial effusion, 4 with spinal cord compression, 2 with intracranial hypertension and 17 with other complications). For some patients, cancer-related complications led to the diagnostic of cancer.

### ICU interventions

The median length of ICU stay was 8 days [3.0–15.0], and median length of hospital stay was 22 days [12.0–32.0].

Eighty-one patients required mechanical ventilation during 6 days [3.0–15.0]. Among these patients, 44 (54%) received iMV at first line, 21 (26%) were initially ventilated with NIV and subsequently required intubation for iMV, and 16 (20%) were only ventilated with NIV. Vasopressors were needed for 61 patients and renal replacement therapies for 12 patients.

### Outcome analysis

Hospital mortality was 60%. ICU, 3 and 6-month mortality rates were, respectively, 47, 67 and 71%. For 50 patients, withdrawal/withhold of life-sustaining therapies (WLTs) were decided after 6 days [2.0–14.5]. Among these patients, 36 patients died in ICU and nine patients died after ICU discharge. Mortality was not different according to the reason of ICU admission (sepsis versus acute respiratory failure, *p* = 0.32) (data not shown).

**Fig. 2:**
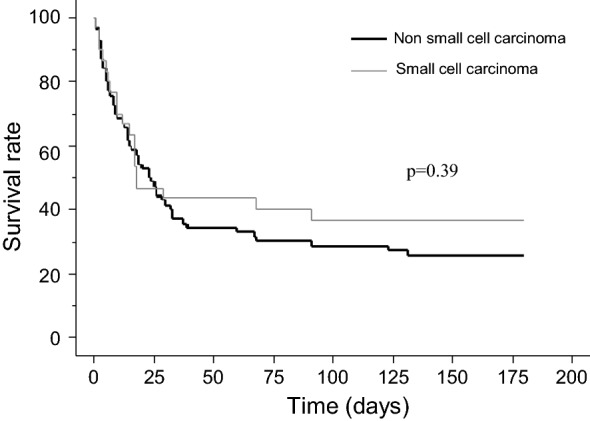
Kaplan–Meier survival curve according to the cancer histology

Mortality of patients differed according to the number of organ failures: hospital mortality was 60% (*n* = 6/10) when patient required only mechanical ventilation, 71% (*n* = 35/49) when patients required mechanical ventilation and vasopressors, and 83% (*n* = 10/12) when they had multiple organ failures. The histological type of cancer was not associated with 6-month mortality (Fig. 2).

Tables 2 and 3 describe patients’ characteristics according to hospital mortality. Metastatic disease (78 vs 57.5%, *p* = 0.040), SOFA score (8 [6.0–14.5] vs 5 [3.5–9.5], *p* = 0.010), SAPS II score (56.5 [46.5–71.0] vs 42.5 [36.5–55.5], *p* = 0.002), need for MV (90 vs 67.5%, *p* = 0.005), need for vasopressors (75 vs 40%, *p* < 0.001) and WLT decisions (75 vs 12.5%, *p* < 0.001) were associated with higher mortality. The use of emergency chemotherapy in ICU, although not statistically significant, showed a trend toward better survival (25 vs 12%, *p* = 0.080).

**Table 2:** Univariate analysis of risk factors associated with hospital mortality

Variables	Survival patients *n* = 40 (40%)	Dead patients *n* = 60 (60%)	*p* value
Age in years, median [IQR]	63 [52.0–71.0]	65.5 [57.0–73.0]	*p* = 0.250
Males, *n* (%)	29 (72.5)	39 (65)	*p* = 0.560
Days since diagnosis, median [IQR]	4 [0–20.5]	7.5 [0–20.5]	*p* = 0.460
Histological type, *n* (%)			*p* = 0.790
SCLC	12 (30)	18 (30)	
NSCLC	28 (70)	42 (70)	
Metastatic disease, *n* (%)	23 (57.5)	47 (78)	*p* = 0.040
Prior chemotherapy, *n* (%)	7 (17.5)	13 (22)	*p* = 0.170
Prior radiotherapy, *n* (%)	3 (7.5)	7 (12)	*p* = 0.250
ECOG-PS, *n* (%)			*p* = 0.920
Low (0–1)	31 (78)	44 (73)	
High	9 (22.5)	15 (25)	
SOFA score, points, median [IQR]	5 [3.5–9.5]	8 [6.0–14.5]	p = 0.010
SAPS II, points, median [IQR]	42.5 [36.5–55.5]	56.5 [46.5–71.0]	*p* = 0.002
Admission reason, *n* (%)			*p* = 0.330
Sepsis	12 (30)	28 (47)	
Respiratory (excluding septic reasons)	21 (52.5)	25 (42)	
Cardiovascular disease	2 (5)	2 (3)	
Neurologic	2 (5)	2 (3)	
Post-CPA	0	2 (3)	
Other	3 (7.5)	1 (2)	

*IQR* interquartile range, *SCLC* small cell lung cancer, *NSCLC* non-small cell lung cancer, *SOFA* Sequential Organ Failure Assessment, *SAPS II* Simplified Acute Physiology Score, version II, *ECOG-PS* Eastern Cooperative Oncology Group performance status, *CPA* cardiopulmonary arrest

**Table 3:** Univariate analysis of ICU’s interventions associated with hospital mortality

Variable	Survival patients *n* = 40 (40%)	Dead patients *n* = 60 (60%)	*p* value
Mechanical ventilation, *n* (%)			*p* = 0.005
No MV	13 (32.5)	6 (10)	
NIV alone	9 (22.5)	7 (12)	
iMV at first line	11 (27.5)	33 (55)	
iMV after NIV	7 (17.5)	14 (23)	
Catecholamines, *n* (%)	16 (40)	45 (75)	*p* < 0.001
Hemodialysis, *n* (%)	2 (5)	10 (17)	*p* = 0.060
Oncologic treatment in ICU, *n* (%)			*p* = 0.080
Chemotherapy	10 (25)	7 (12)	
WLT	5 (12.5)	45 (75)	*p* < 0.001

*ICU* intensive care unit, *MV* mechanical ventilation, *iMV* invasive mechanical ventilation, *NIV* noninvasive ventilation, *WLTs* withdrawal/withhold life-sustaining therapies

Factors independently associated with hospital mortality are reported in Table 4.

**Table 4:** Multivariate analysis of risk factors independently associated with hospital mortality

Variable	OR [95%CI]	*p* value
Metastatic disease	4.22 [1.4–12.4]	*p* = 0.008
Chemotherapy in ICU	0.23 [0.07–0.81]	*p* = 0.020
Vasopressor	2.67 [0.8–8.9]	*p* = 0.100
Mechanical ventilation		
NIV alone or no mechanical ventilation	1	
iMV after NIV	4.18 [0.88–19.9]	*p* = 0.070
iMV at first line	4.2 [1.11–16.2]	*p* = 0.030

*OR* odds ratio, *CI* confidence interval, *iMV* invasive mechanical ventilation, *NIV* noninvasive ventilation

### Cancer treatment

Twenty patients received chemotherapy as front-line treatment of their cancer; 10 were treated with radiotherapy. (Six among these ten received combined regimens of radiotherapy and chemotherapy.) The details of oncologic treatments were not available in the databases.

Seventeen patients received chemotherapy during ICU stay, mostly presenting with SCLC.

In total, most of the patients did not receive cancer treatment before ICU discharge and chemotherapy could eventually be decided after ICU stay.

### Outcome of patients who received emergency chemotherapy in ICU

Seventeen patients received emergency chemotherapy while in ICU. Mostly they had small cell lung cancer (SCLC) (*n* = 11), performance status was good (ECOG-PS < 2 for 14 patients) and they had few comorbidities (CCI of 0 [0–2.0]). SOFA and SAPSII scores were, respectively, of 7 [3.0–8.0] and 51 [39.0–59.0]. None of these patients had received previous oncologic treatment for lung cancer. Except one, they all required MV during ICU stay. For 15 patients (88%), the reason for emergency chemotherapy was cancer-related severe acute complications. The most frequent complications were airway obstruction related to cancer (*n* = 10/15, 67%) and/or pleural effusion (*n* = 5/15, 33%). ICU, hospital, 3- and 6-month mortality rate were, respectively, 29, 41, 53 and 59%. Among the ten patients discharged from hospital (including eight with SCLC), eight patients (80%) were alive at 3 months (all SCLC) and seven patients (70%) were alive at 6 months. For those patients who received MV and vasopressors, mortality rate was 46% and for those with multiple organ failure mortality rates was 50%.

## Discussion

In this multicentric, retrospective study, patients with newly diagnosed lung cancer admitted to ICU had acceptable ICU and hospital mortality rates of, respectively, 47 and 60%. However, mortality rates at three and 6 months remained substantially high (respectively, 67 and 71%). As expected, mortality rates rose with the severity of acute illness. Although mortality for patients who required only iMV was 60%, it reached 83% for patients with multiple organ failure. Those results were consistent with previous studies [12, 14, 15, 24] and could raise questions about the futility of intensive care for these last patients. In our study, decision to withhold or withdrawal life-sustaining therapies occurred for half of the patients with a high rate of mortality. (Seventy-two percentage of these patients died in ICU and 90% in the hospital.) Decreasing the number of unnecessary aggressive care is a major concern in this population, especially with the increasing number of patients treated for advanced cancer and therefore the number of patients with cancer-related emergencies [25]. The decisional process should include intensivists, oncologists and palliative care services. Triage criteria for this specific population of patients are still imperfect [9], and prognosis factors have been pursued to select patients who would benefit the most from intensive cares. Moreover, triage criteria should be frequently reassessed according to new treatment, and survival improvement in that setting [26].

Various factors are associated with mortality in the studies [27]. Besides organ failure related to acute disease, we found in our study two factors independently associated with hospital outcome and related to cancer characteristics. Metastatic disease was associated with mortality (*p* = 0.003), and the administration of chemotherapy during ICU stay was associated with survival (*p* = 0.020). In contrast to other previous studies [11, 13, 18], performance status was not associated with hospital mortality. This result could be related to the proportion of patients with good performance status in our cohort and to the analysis of hospital mortality only and not long-term outcome. Moreover, other prognosis factors have been described in oncology for advanced cancer patients, such as anorexia–cachexia syndrome, delirium, leukocytosis, lymphocytopenia, levels of C-reactive protein [28] or combinations of criteria, including Karnofsky index, number of metastatic sites, levels of serum albumin and lactate dehydrogenase (LDH) concentration [29]. They should be assessed for critically ill patients with inaugural diagnosis. Prospective large multicentric studies or meta-analysis is needed.

Our study added interesting data about critically ill lung cancer patients at the diagnosis of their malignancy. Studies concerning newly diagnosed lung cancer patients with life-threatening complications remained scarce [30, 31]. We defined new diagnosis as diagnosis within the month of ICU admission so that patients would not have received more than one line of treatment. Our results appear similar to recent studies on critically ill lung cancer patients, at different times of their disease [12, 18, 19].

A major strength of our study was that 17 patients were able to receive chemotherapy during their ICU stay. These patients presented mostly with SCLC. Chemotherapy was prescribed during ICU stay for 88% of patients because of cancer-related complications. Among these patients, ICU, hospital, 3- and 6-month mortality rates were, respectively, 29, 41, 53 and 59%. Receiving chemotherapy in ICU was independently associated with survival (*p* = 0.020). This suggested that rescue chemotherapy in ICU is feasible in selected patients and had a positive outcome. It also suggests that the tumor’s chemo-sensitivity is an important factor that should be taken into account in the decision of admitting patients in ICU. Because of their high response rate to chemotherapy [32], patients with SCLC remained good candidates for ICU admission at diagnosis. These results are consistent the study of Zerbib et al. [33] in which SCLC has been identified as an independent predictor of hospital survival for patients receiving chemotherapy in ICU for organ failure related to solid neoplasms. In another study by Chen et al. [30], ICU and hospital survival were better for patients who received either chemotherapy or epidermal growth factor receptor-tyrosine kinase inhibitor (EGFR-TKI) therapy compared with those receiving best supportive care. They also found that ICU survival was independently associated with the use of mechanical ventilation, which is different from our results. This difference might be explained by the particular population of the study composed by a high number of patient treated with EGFR-TKI with usually high rate of good and quick response to treatment [34]. Newly diagnosed lung cancer patients, especially those with high sensitivity to anticancer treatment admitted to the ICU for cancer-related complications, appear as a specific subgroup of patient who might benefit from invasive cares. Other studies are warranted to confirm those results, to explore the type and timing of anticancer therapy for this subpopulation, and data must be considered with caution over time since therapeutic advances in oncology are substantial. However, the increasing number of treated patients would lead to high rate of critical care admission. The decision for ICU admission, but also the assessment of the goals of care during ICU stay, should include intensivist, oncologist and palliative care physician to improve the best care for those patients [9]. Studies are needed to improve the best model of delivering care in that setting.

The present study has several limitations. First, it was retrospective, monocentric and focused on a small number of patients admitted to ICU. Although all the patients were diagnosed with lung cancer within 1 month and had a good performance status before ICU admission, the possibility of cancer treatment after complications leading to ICU admission could be small [35]. There were no details concerning triage decisions, and we could not analyze the outcome of patients who were referred, but not admitted, to the ICU. Second, the choice of the severity scores that has been made in this study can be debated. We use the SOFA and the SAPSII scores, but no differences have been clearly found between the different existing scores [36, 37] and others such as the Acute Physiology and Chronic Health Evaluation (APACHE) could have been used [38] in the specific population of cancer patients. Third, only a small number of patients received chemotherapy. There was a lack of details regarding the oncologic treatments received before ICU admission and type of chemotherapy regimens used during ICU stay, which might have an impact on the outcome. Tolerance and treatment-related toxicities of chemotherapy have not been recorded and were other important issues. Other treatments for non-small cell lung cancer (NSCLC), such as targeted therapies, were not analyzed in this study. However, some studies [30] confirmed improvements in the outcome for specific patients admitted to ICU with mutated NSCLC. Fourth, we did not describe outcome according to the metastatic stage. However, for ICU patients, number of metastatic site was not related to outcome in recent study [30]. Also, for some patients with diagnosis performed during ICU, metastatic stage was not completely known at ICU admission. Lastly, although 40% of patients were still alive at hospital discharge, we do not have any information about the quality of life and the possibilities to receive further oncologic treatments.

In conclusion, this multinational study showed that ICU management was appropriate for newly diagnosed, unresectable lung cancer patients. Nevertheless, tumor’s characteristics, number of organ dysfunctions and types of intensive interventions should be taken into account before admitting these patients in ICU. Metastatic disease and need for immediate invasive iMV were associated with mortality, and mortality rose with the severity of acute illness. The tumor’s chemo-sensitivity should also be estimated since rescue chemotherapy in ICU was associated with survival and should be proposed for selected patients, especially for those with cancer-related complications.

## Appendix

### Participating centers for the LUCCA database

#### Argentina

Instituto Medico Especializado Alexander Fleming, Buenos Aires (Célica Irrazábal, Pierina Bachetti).

#### Brazil

Instituto Nacional de Câncer, Rio de Janeiro (Vicente C. Souza-Dantas, Mauro M. Zamboni, Aureliano Sousa).

Hospital A. C. Camargo, São Paulo (Bruno F. C. Almeida, Lúcio S. Santos, Pedro Caruso), Fundação Pio XII - Hospital de Câncer de Barretos, Barretos (Ulysses V. A. Silva).

Hospital Sírio Libanês, São Paulo (Luciano C. P. Azevedo, Guilherme P. P. Schettino).

Vitória Apart Hospital, Vitória (Cláudio Piras, Stéphanie B. Piras, Albano S. M. T. Silva).

#### Chile

Hospital Clinico Universidad de Chile, Santiago (Eduado Tobar, Nivia Estuardo).

#### France

Institut Gustave Roussy, Villejuif (François Blot, Bruno Raynard).

Hôpital Tenon, Paris (Antoine Parrot).

Hospices Civils de Lyon, Centre Hospitalier Lyon Sud, Lyon (Florent Wallet).

Institut Mutualiste Montsouris, Paris (Christian Lamer).

Groupe Hospitalier Pitié Salpêtrière, Paris (Alexandre Duguet, Alexandre Demoule, Julie Delemazure, Julien Mayaux, Thomas Similowski).

Hôpital A. Michallon, CHU de Grenoble, Grenoble (Surgical ICU: Michel Durand, Geraldine Dessertaine, Pr Jean François Payen; Medical ICU: Anne-Claire Toffart, Jean-François Timsit).

Hôpital de la Croix-Rousse, Lyon (Gael Bourdin, Claude Guerin).

Hôpital Hôtel Dieu de Paris, Paris (Antoine Rabbat; Aurélie Lefebvre).

Hôpital Saint-Louis, Paris (Élie Azoulay).

#### United Kingdom

Royal Marsden Hospital, London (Natalie Pattison).

Royal Brompton NHS Foundation Trust, London (Natalie Pattison).

#### Uruguay

Hospital Maciel, Montevideo (Gastón Burghi).

Asocianción Española Primera de Socorros Mutuos, Montevideo (Gastón Burghi).

Hospital de Clínicas, Montevideo (Gastón Burghi).

## Abbreviations

CCI: Charlson Comorbidity Index
ECOG-PS: Eastern Cooperative Oncology Group performance status
ICU: intensive care unit
iMV: invasive mechanical ventilation
LDH: lactate dehydrogenase
MV: mechanical ventilation
NIV: noninvasive mechanical ventilation
NSCLC: non-small cell lung cancer
SAPSII: Simplified Acute Physiology Score
SCLC: small cell lung cancer
SOFA: Sequential Organ Failure Assessment
WLTs: withdrawal/withhold life-sustaining therapies
